# Caracterização Histopatológica das Lesões Valvares Mitrais em Pacientes com Cardiopatia Reumática: A Inflamação Também é Responsável pela Progressão da Valvopatia Crônica?

**DOI:** 10.36660/abc.20201244

**Published:** 2021-03-03

**Affiliations:** 1 Universidade de São Paulo Faculdade de Medicina Hospital das Clínicas São PauloSP Brasil Unidade Clínica de Valvopatias do Instituto do Coração do Hospital das Clínicas da Faculdade de Medicina da Universidade de São Paulo (InCor HC FMUSP), São Paulo, SP - Brasil.

**Keywords:** Insuficiência da Valva Mitral/cirurgia, Cardiopatia Reumática/diagnóstico, Inflamação, Fibrose, Epidemiologia

A febre reumática (FR) e a doença reumática cardíaca (DRC) continuam a ser altamente prevalentes, afetando aproximadamente 40 milhões de pessoas, principalmente em países de baixa e média renda, mas também em populações marginalizadas em países desenvolvidos.^1^^,^^2^ Os dados brasileiros sobre prevalência da FR são escassos principalmente devido a (a) dificuldades no diagnóstico de FR aguda, (b) custo do rastreamento de DRC e (c) o fato de que dados relatados sobre cirurgia ou óbito podem representar incidência da FR de 2 décadas atrás. A consequência mais temida da FR são as valvopatias crônicas, que levam à piora na qualidade de vida, hospitalizações e necessidade de procedimento cirúrgico, principalmente em indivíduos jovens.^3^^,^^4^ Portanto, há uma necessidade urgente de melhor compreender os fatores que influenciam a progressão da doença cardíaca valvar (DCV). Nesse contexto, Gomes et al.^5^ estudaram as alterações histopatológicas nas válvulas mitrais de pacientes com DRC submetidos à troca valvar mitral.

A inflamação é um dos principais componentes da DCV em pacientes com FR. Na fase aguda da infecção, os achados histopatológicos consistem na presença de densos infiltrados inflamatórios valvares e nódulos de Aschoff, caracterizados como degeneração fibrinoide do colágeno circundado por linfócitos, macrófagos, células gigantes, plasmócitos e fibroblastos em paliçada.^6^^–^^9^ A DRC crônica geralmente se apresenta com calcificação das cúspides, fibrose e fusão comissural. Entretanto, o papel da inflamação na DCV crônica ainda está em estudo.

Gomes et al.^5^ examinaram 60 válvulas mitrais explantadas, 40 de etiologia reumática (53 ± 13 anos; 90% mulheres) e 20 de um grupo controle constituído de pacientes submetidos a transplante cardíaco (50 ± 12 anos; 70% homens). Quando comparados ao grupo controle, os pacientes reumáticos apresentaram mais fibrose, neoangiogênese, calcificação e inflamação de intensidade moderada ou importante. A presença de um processo inflamatório crônico ativo na válvula mitral requer um melhor entendimento da progressão da degeneração reumática da DCV. Além disso, esses achados sugerem que as opções terapêuticas médicas direcionadas à inflamação da válvula podem retardar a progressão da doença nos estágios finais da DCV. Entretanto, duas considerações importantes em relação ao desenho do estudo devem ser feitas. Primeiro, os pacientes do estudo e o grupo controle não foram pareados para vários parâmetros clínicos e ecocardiográficos. Em segundo lugar, não é possível descartar a possibilidade de que o processo inflamatório crônico seja consequência do estresse hemodinâmico na valva lesada e não relacionado à DRC. Para esse propósito, um grupo controle deveria ser constituído de pacientes com valvopatia anatomicamente importante não-reumática, como prolapso de válvula mitral.

Além disso, os autores também compararam os achados histopatológicos de acordo com a lesão valvar predominante, isto é, estenose e regurgitação. Pacientes com estenose mitral apresentaram maior calcificação das cúspides, como esperado. No entanto, os pacientes com maior grau de inflamação apresentaram maior área valvar mitral. Esses achados geram duas hipóteses sobre o padrão de lesão valvar:

1. A inflamação leva à regurgitação mitral: alguns pacientes apresentam mais inflamação valvar, maior área valvar mitral e, portanto, têm um predomínio de regurgitação mitral;
2. A inflamação leva à estenose mitral: e a inflamação é reduzida à medida que a calcificação aparece. Portanto, pacientes com menos inflamação têm áreas valvares menores.

Infelizmente, a FR ainda é uma doença marginalizada, com poucos estudos sobre sua fisiopatologia. O estudo realizado por Gomes et al.^5^ forneceu novas informações importantes sobre a inflamação na DRC crônica. Entretanto, mais pesquisas são necessárias para melhor entender a progressão da DCV, os padrões de lesão valvar e, portanto, estabelecer novas opções de tratamento.
