# Supplementary material for: Caracterização Histopatológica das Lesões Valvares Mitrais em Pacientes com Cardiopatia Reumática: A Inflamação Também é Responsável pela Progressão da Valvopatia Crônica?
Source: Arq Bras Cardiol. 2021 Mar 3;116(3):413–4. [Article in Portuguese] doi: 10.36660/abc.20201244 (PMC8159542; doi:10.36660/abc.20201244)
Supplement: Translation

# Translation: Histopathological Characterization of Mitral Valvular Lesions in Patients With Rheumatic Heart Disease: Is Inflammation Also to Blame for Chronic Valvular Heart Disease Progression?

**Affiliations:** 1 São PauloSP Brazil Unidade Clínica de Valvopatias do Instituto do Coração do Hospital das Clínicas da Faculdade de Medicina da Universidade de São Paulo (InCor HC FMUSP), São Paulo, SP - Brazil.

**Keywords:** Mitral Valve Insufficiency/surgery, Rheumatic Heart Disease/diagnosis, Inflammation, Fibrosis, Epidemiology

Rheumatic fever (RF) and rheumatic heart disease (RHD) remain highly prevalent, affecting approximately 40 million people, mostly in low- and middle-income countries, but also in select marginalized populations in higher-income countries.^1^^,^^2^ Brazilian data regarding RF prevalence is scarce mainly due to (a) difficulties in acute RF diagnosis, (b) cost of RHD screening and (c) the fact that reported data on surgery or death may represent RF incidence from 2 decades ago. The most feared consequence of RF is valvular heart disease, which leads to a worsening in the quality of life, hospitalizations and need for surgical procedure, primarily in young people.^3^^,^^4^ Therefore, there is an urgent need to better understand the factors that influence valvular heart disease (VHD) progression. In this context, Gomes et al.^5^ studied the histopathological changes in mitral valves of patients with RHD undergoing mitral valve replacement.

Inflammation in one of the key components of VHD in patients with RF. In the acute stage of infection, the histopathological findings are the presence of dense valvular inflammatory infiltrates and Aschoff nodules, characterized as collagen fibrinoid degeneration surrounded by lymphocytes, macrophages, giant cells, plasma cells, and palisades of fibroblasts.^6^^–^^9^ Chronic RHD usually presents with cuspid calcification, fibrosis and commissural fusion. However, the role of inflammation in chronic VHD is still under study.

Gomes et al.^5^ examined 60 explanted mitral valves, 40 of rheumatic etiology (53 ± 13 years; 90% females) and 20 from a control group consisting of patients submitted to heart transplant (50 ± 12 years; 70% males). When compared to the control group, rheumatic patients had more fibrosis, neoangiogenesis, calcification and moderate- or severe-intensity inflammation. The presence of an active chronic inflammatory process in the mitral valve requires a better understanding of rheumatic VHD degeneration progression. Besides, these findings suggest that medical therapeutic options targeting valve inflammation may slow the disease progression in the late stages of VHD. However, two important considerations regarding the study design must be pointed out. First, the study patients and the control group were not matched for several clinical and echocardiographic parameters. Second, it is not possible to rule out that the chronic inflammatory process was a consequence of the hemodynamic stress in the injured valve, and unrelated to the RHD. For this purpose, a control group should consist of patients with non-rheumatic severe valvular heart disease, such as mitral valve prolapse.

In addition, the authors also compared the histopathological findings according to the predominant valve lesion, i.e. stenosis and regurgitation. Mitral stenosis patients had more cuspid calcification, as expected. However, patients with a higher degree of inflammation had a larger mitral valve area. These findings generate 2 hypotheses about the valve injury pattern:

1. Inflammation leads to mitral regurgitation: some patients have more valve inflammation, a larger mitral valve area and thus have a predominance of mitral regurgitation;
2. Inflammation leads to mitral stenosis: as calcification appears, the inflammation is reduced. Hence, patients with less inflammation have smaller valve areas.

Unfortunately, RF is still a marginalized disease with few studies on its pathophysiology. The study carried out by Gomes et al.^5^ provided new important information about inflammation in chronic RHD. However, further research is required to better understand VHD progression, valve injury patterns and, therefore, to establish new treatment options.
